# Temporomandibular Myofacial Pain Treated with Botulinum Toxin Injection

**DOI:** 10.3390/toxins7082791

**Published:** 2015-07-24

**Authors:** Niv Mor, Christropher Tang, Andrew Blitzer

**Affiliations:** 1 Mount Sinai Roosevelt Hospital, Department of Otolaryngology—Head and Neck Surgery, New York, NY 10019, USA; E-Mails: christophergtang@gmail.com (C.T.); ab1136@aol.com (A.B.); 2 Head and Neck Surgical Group—New York Center for Voice and Swallowing Disorders, New York, NY 10019, USA

**Keywords:** temporomandibular joint, temporomandibular disorders, botulinum toxin, myofacial pain

## Abstract

This article reviews the diagnoses and treatment of temporomandibular disorders (TMD) and outlines of the role of botulinum toxin (BoNT) in the treatment of myofacial TMD. This manuscript includes a brief history of the use of BoNT in the treatment of pain, the mechanism of action of BoNT, and the techniques for injections, adverse effects and contraindications when using BoNT to treat mayofacial pain caused by TMD.

## 1. Introduction

The temporomandibular joint (TMJ) is a hinged synovial joint that connects the mandible to the temporal bone at the skull base, and the posterior boarder of the TMJ is the anterior boarder of the external auditory canal. The TMJ is one of the few synovial joints with an articular disc and it functions as both a hinge joint and a sliding joint. It is therefore classified as a ginglymoarthrodial joint. Adduction of the mandible, or mouth closing, is performed by the actions of the masseter, temporalis, and medial pterygoid muscles. Abduction, or mouth opening, is performed by the lateral pterygoids and digastric musculature. The lateral deviation occurs by the action of the contralateral lateral pterygoid muscles, and protrusion of the mandible occurs when right and left lateral pterygoid muscles contract simultaneously.

Temporomandibular disorder (TMD) is a nonspecific term used to describe orthopedic and myofascial disorders that affect the TMJ. The prevalence of TMD is between 30% and 44%, with up to 25% of the population seeking professional care for TMD [1]. Symptoms are commonly related to pain surrounding the joint and may include headache, periauricular pain, neck pain, decreased jaw excursion, jaw locking, and noise at the joint with movement. In general TMD is divided into myofacial TMD or arthrogenic TMD. Myofacial TMD is associated with the pain from hyperfunctioning muscles of mastication leading to chronic myositis. In contrast, arthrogenic TMD is associated intracapsular pathology with pain at the level of the joint itself.

## 2. Diagnosing Temporomandibular Disorders

The diagnosis of TMD is based on history and physical exam findings. Patients should be asked about nighttime bruxism, jaw soreness, morning headaches, use of mouth orthodonitics, or history of trauma. Questions regarding the patient’s personal habits and diet should be evaluated to reduce behaviors that may place additional stress on the TMJ, like frequent gum chewing. Symptoms of depression, anxiety or recent stressors should also be assessed as these conditions often lead to unconsciously clench.

Signs and symptoms of TMD may include joint or muscular pain with or without jaw opening/closing, limited jaw movement, cracking or popping sounds at the TMJ with movement, or headaches. Patients often present with otalgia, ear fullness or tinnitus which is referred from the anterior external auditory canal, and is a shared boarder with the posterior TMJ [2]. In addition otalgia may be referred from muscles of mastication as some middle ear muscles (tensor tympani and tensor palatini) are also innervated by the trigeminal nerve [3]. Some patients with TMD have complained of unilateral and boring orbital or periorbital pain, which could be neurogenic or due to hyperfunctioning temporalis muscles [4,5,6].

Imaging studies are rarely obtained at the initial visit, and are generally used only when there is dysfunction of the articular disc or other disorders that may complicate the diagnosis like rheumatoid arthritis, osteoarthritis or trauma [7]. If imaging is obtained, a panoramic plain film of the jaws is usually the initial imaging modality with maxillofacial computed tomography (CT) or magnetic resonance imaging (MRI) of the temporomandibular joints obtained when a more detailed anatomical examination is necessary.

In 1992, The Research Diagnostic Criteria for Temporomandibular Disorders (RDC/TMD) was established to standardize the clinical assessment of patients with TMD. RDC/TMD classifies TMD into two categories (Axis 1 and Axis 2). Axis 1 focuses on physical symptoms like myofascial pain, temporomandibular disc displacement, and joint disorders including arthralgias, arthritis, and arthrosis. Axis 2 focuses on quality of life and psychosocial factors like depression, pain related disability and behavioral adaptions [8]. Today, RDC/TMD is used mainly for research purposes, and is rarely used in clinical diagnosis.

## 3. Treating Temporomandibular Disorders

Treatments for TMD range from nonpharmacologic therapy, conservative pharmicothearpy and open surgery. The initial management consists of nonpharmacologic management and typically includes avoiding triggers, adjusting diet, pain management, physical therapy and warm compress. Patients with bruxism should be referred to a dentist and can be evaluated for an occlusal splint. Systemic pharmacotherapy for TMD may be used as adjuctive therapy and includes anti-inflammatory agents, muscle relaxants, analgesics and in some cases tricyclic antidepressants [9,10,11,12]. Other less conventional approaches include acupuncture, biofeedback or cognitive behavioral therapy [13,14,15]. Selected patients with arthrogenic TMD may benefit from intra-articular corticosteroid injections, arthrocentesis or arthroscopic surgery. Despite the effectiveness of analgesic pain medications, response to opioid therapy is often incomplete with approximately three quarters of patients suffering from persistent pain [16]. Botulinum toxin (BoNT) injection has therefore become an attractive choice as adjuvant therapy in patients with myofacial TMD who do not achieve a complete response with conservative management and pharmacotherapy.

## 4. Botulinum Toxin

The first indication that BoNT could be useful for treating pain was observed from anecdotal reports of patients treated for hyperfunctional facial lines who reported reduced frequency and severity of headache [17]. Soon thereafter, the pain-relieving effect of BoNT was reported during the treatment of oromandibular dystonia and cervical dystonia [18,19,20,21,22]. Today, BoNT is used for pain relief in numerous conditions including tension headaches, migraine headaches, post-herpetic neuralgia and myofacial TMD [23,24,25,26,27].

## 5. Botulinum Toxin Mechanism of Action

BoNT is a 150-Kilodalton exotoxin produced from clostridium botulinum, whose action is mediated through the cleavage of docking proteins that are responsible for membrane fusion of pre-synaptic vesicles. Type A bolulinum toxin (BoNT-A) cleaves the membrane associated protein “synaptosomal-associated protein 25” (SNAP-25) which is a member of the “soluble *N*-ethylmaleimide sensitive factor attachment protein receptor protein (SNARE). Type B botulinum toxin (BoNT-B) cleaves synaptobravin, which is part of the vesicular-associated membrane protein (VAMP). Cleavage of these docking proteins leads to muscle weakens by inhibition of acetylcholine (Ach) release at the neuromuscular junction. In addition to blocking the activity of muscles, BoNT has been known to inhibit the release for mediators from numerous secretory glands including salivary glands, sweat glands and nasal mucosa [28,29,30,31,32,33,34,35,36,37,38,39,40].

Chronic local muscular contracture has been known to cause inflammation and localized muscular hypoxia leading to chronic myofascial pain [41]. However, the analgesic effect of BoNT comes from more than just stress relief to the musculature. BoNT has a direct effect on nociceptors and the parasympathetic nervous system [42]. Inflammatory mediators like calcitonin gene-related peptide (CGRP), substance P and glutamate are also regulated by SNARE and VAMP docking proteins and, their release is inhibited by BoNT. The effect of BoNT also effects pain processing which reduces central pain sensitization, the driving mechanism of chronic pain. A-delta sensory fibers, which mediate acute pain, and A-beta fibers, which mediate touch and pressure, are not mediated by neuropeptide release and are unaffected by BoNT. Thus, BoNT does not interfere with the perception of acute pain nor does it cause local anesthesia.

## 6. Botulinum Toxin Injection Technique

The most commonly affected muscles are the temporalis muscle, masseter muscle and lateral pterigoid muscles. The temporalis muscle and masseter muscle are almost always involved and usually manifest as direct muscle pain. Lateral pterygoids involvement usually manifests as buccal pain, lateral jaw deviation or bruxism [43].

Although Botox (onabotulinumtoxinA), Dysport (abobotulinumtoxinA), Xeomin (incobotulinumtoxinA) or Myobloc (rimabotulinumtoxinB) have been approved for use by the U.S. Food and Drug Administration (FDA) for various other conditions including bladder dysfunction, chronic migraine, upper limb spasticity, cervical dystonia, primary axillary hyperhidrosis, blepharospasm, strabismus, glabellar rhytides and lateral canthal rhytides, no product is currently approved by the FDA to treat TMD [44]. Furthermore, there is no established method to calculate equivalent doses between different BoNT products. The potency per unit of one product is not interchangeable with other preparations and each may have different safety and efficacy characteristics. It is therefore important that clinicians be familiar with the various formulations prior to their use [45].

**Figure 1:**
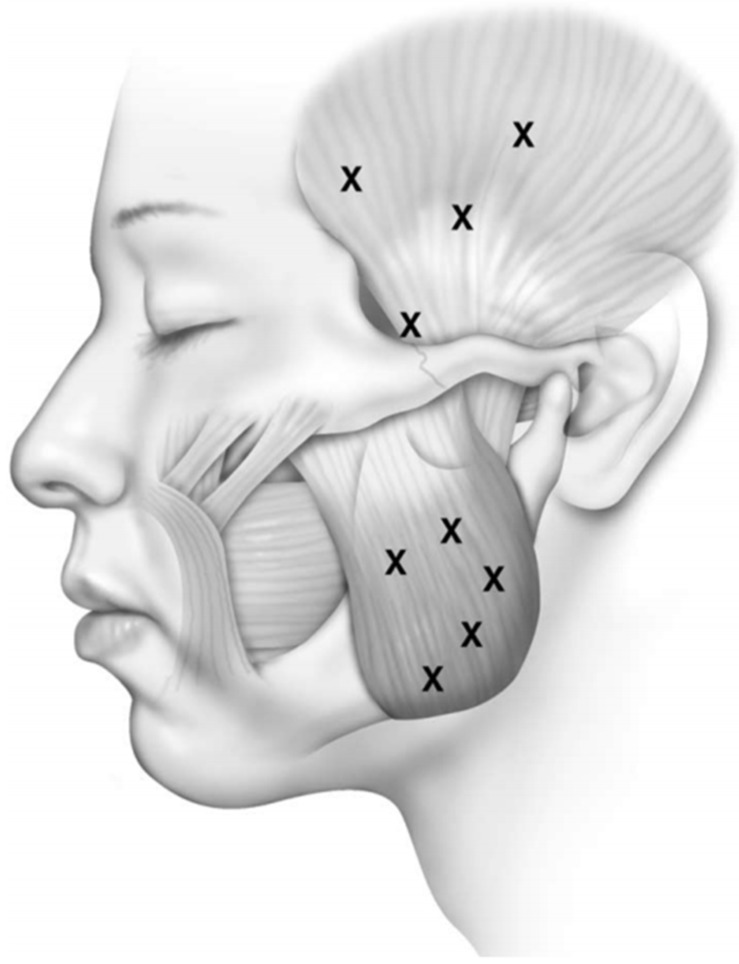
Facial diagram of trancutaneous needle placement for injection of botulinum toxin to the temporalis and masseter muscles. Reproduced from [46]. Copyright Elsevier Inc., 2004.

**Figure 2:**
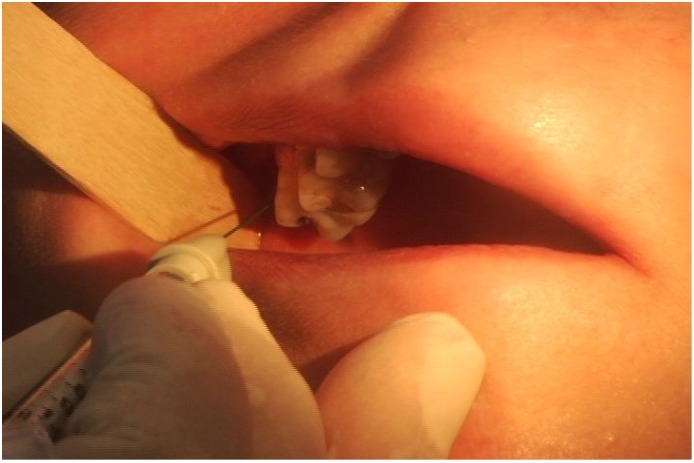
Photograph of intraoral EMG guided injection of botulinum toxin to the lateral pterygoid muscle (EMG electromyography). Reproduced from [47]. Copyright Thieme Medical Publishers, Inc., 2012.

We performe injections under the guidance of electromyography (EMG) using a 27-gauge monopolar electrode injection needle. The temporalis and masseter muscles are injected transcutaneously (Figure 1). Asking the patient to clench their teeth will aid in precise EMG localization. Identification of the lateral pterygoid muscle is done intraorally with the EMG needle placed between the pterygoid plate and the coronoid process of the mandible (Figure 2). In this fashion injection is achieved along the length of the muscle, and confirmation of intramuscular injection is obtained by robust EMG signaling with lateral jaw movements.

We typically use a concentration of 2.5–5.0 units per 0.1 mL of Botox witha starting dose of 10–25 units for each temporalis muscle, 25–50 units to the masseter muscles and 7.5–10 units to the lateral pterygoids. Subsequent doses are individualized and are based on the patients’ response. Botox diffuses to about 1 cm at each injection site, and affected areas may be left untreated if an inadequate number of sites are infiltrated within a single muscle group. To avoid an incomplete response, we advocate using lower concentrations at multiple sites with larger injection volumes. 

## 7. Therapeutic Effects of Botulinum Toxin

Approximately 3-4 weeks after the initial injection, patients will return for reevaluation and documentation any adverse effects and/or suboptimal responses. Some patients may require a booster injection at that time. Additional BoNT injections are directed by the patients’ history and clinical examination, and pain diaries are a useful guide to help in patient directed therapy. It may take several weeks before patients experience the maximum pain relief from BoNT. Althoguth the typical duration of efficacy is 12 weeks, we have noted tremendous variability among patients with respect to optimal dosing frequency. Some patients experience relief well beyond the predicted pharmacokinetic duration of the drug supporting the possibility that BoNT does not strictly act at the periphery and may be involved in neuromodulation at the level of the central nervous system. It is important to note that an individual patient’s response to toxin may change over time, which further supports the importance of patient directed dosing [48].

## 8. Adverse Effects of Botulinum Toxin

BoNT injection for TMD is administered in relatively small doses and way below the estimated lethal dose of approximately 3000 units. Because of the relative low dosing profile, adverse effects are uncommon and often mild. Difficulty chewing is the most common adverse effect, which results from local muscle weakness and is usually dose dependent. Muscle atrophy may result in cosmetic alterations and is another risk of the procedure. Higher volume of BoNT increased the risk of diffusion of toxin to nearby areas which may cause brow ptosis, blepharoptosis or diplopia if the temporalis muscle is injected too close to the orbit. Facial asymmetry may result if the masseter muscle is injected too close to the zygomaticus major [39,41,47,49]. We therefore recommend directing the needle laterally when injecting to minimize this diffusion effect. Dry mouth may occur if BoNT is injected into the parotid gland. Flu-like syndromes rarely occur and and are usually of brief duration. In addition, with any injection there is an inherent risk associated with a needle puncture, such as bruising and local tenderness.

## 9. Contraindications to Botulinum Toxin

Contraindications to BoNT use include a known allergy to BoNT, active inflammation or infection at the proposed injection site, pregnancy, breast-feeding, or chronic degenerative neuromuscular disorders like amyotrophic lateral sclerosis, myasthenia gravis, Lambert-Eaton syndrome, muscular dystrophy or multiple sclerosis. Patients taking aminoglycoside antibiotics should not receive BoNT injections because this class of antibiotics may interfere with the neuromuscular transmission of toxin and potentiate the effect of BoNT [50].

## 10. Botulinum Toxin Outcomes

Numerous studies have shown that BoNT provides long-term relief of myofacial TMD by decreasing the intensity, frequency, and duration of recurrent episodes. Freund *et al* reported on 46 patients with TMD treated with 150 units of BoNT-A to the masseters and termporalis muscles. They found significant reductions in their subjective and objective pain scores, and all patients with restricted mouth opening had some degree of improved range of motion. They also reported successful treatment various conditions that fall under the general category of TMD such as bruxism and clenching, oromandibular dystonias, trismus, masseter and temporalis hypertrophy, and headaches [51]. In an open-label study of roughly 100 patients with TMD the senior author found a 70% response rate to BoNT-A injections to the masseter, temporalis and lateral pterygoid muscles. Response was defined as a 50% or greater reduction of subjective pain and/or frequency of pain [46]. In a multicenter randomized double-blind, placebo controlled fixed dose study (50 units Botox to each masseter muscle and 25 units Botox to each temporalis muscle), reduction of subjective pain and tenderness to palpation was greatest at eight weeks following injections [52]. The authors of this study also noted a decrease in the average daily use of pain medication over the 16 week duration of this study.

## 11. Summary

Temporomandibular disorder is a common cause of chronic facial pain and is known to interfere with personal relations, professional duties, and overall quality of life. Appropriate diagnosis allows physicians to identify the disorder and initiate an effective therapeutic plan. Botulinum toxin has provided long-term relief of TMD by reducing the intensity, frequency, and duration of recurrent episodes. Adverse effects from BoNT injections are uncommon, mild and transient making it an attractive option for adjunctive therapy for myofacial TMD in patients who have failed initial conservative therapy and systemic pharmacotherapy.
